# Cryogel Scaffold-Mediated Delivery of Adipose-Derived Stem Cells Promotes Healing in Murine Model of Atrophic Non-Union

**DOI:** 10.3389/fbioe.2022.851904

**Published:** 2022-05-05

**Authors:** Katherine R. Hixon, Dakota B. Katz, Jennifer A. McKenzie, Anna N. Miller, Farshid Guilak, Matthew J. Silva

**Affiliations:** ^1^ Department of Orthopaedic Surgery, Musculoskeletal Research Center, Washington University, St. Louis, MO, United States; ^2^ Thayer School of Engineering, Dartmouth College, Hanover, NH, United States; ^3^ Department of Biomedical Engineering, Washington University, St. Louis, MO, United States; ^4^ Center of Regenerative Medicine, Washington University, St. Louis, MO, United States; ^5^ Shriners Hospitals for Children—St. Louis, St. Louis, MO, United States

**Keywords:** animal models, fracture healing, tissue engineering, cryogels, BMP-2

## Abstract

Non-union is defined as the permanent failure of a bone to heal and occurs clinically in 5% of fractures. Atrophic non-unions, characterized by absent/minimal callus formation, are poorly understood and difficult to treat. We recently demonstrated a novel murine model of atrophic non-union in the 3.6Col1A1-tk (Col1-tk) mouse, wherein dosing with the nucleoside analog ganciclovir (GCV) was used to deplete proliferating osteoprogenitor cells, leading to a radiographic and biomechanical non-union after the mid-shaft femur fracture. Using this Col1-tk atrophic non-union model, we hypothesized that the scaffold-mediated lentiviral delivery of doxycycline-inducible BMP-2 transgenes would induce osteogenesis at the fracture site. Cryogel scaffolds were used as a vehicle for GFP+ and BMP-2+ cell delivery to the site of non-union. Cryogel scaffolds were biofabricated through the cross-linking of a chitosan–gelatin polymer solution at subzero temperatures, which results in a macroporous, spongy structure that may be advantageous for a bone regeneration application. Murine adipose-derived stem cells were seeded onto the cryogel scaffolds, where they underwent lentiviral transduction. Following the establishment of atrophic non-unions in the femurs of Col1-tk mice (4 weeks post-fracture), transduced, seeded scaffolds were surgically placed around the site of non-union, and the animals were given doxycycline water to induce BMP-2 production. Controls included GFP+ cells on the cryogel scaffolds, acellular scaffolds, and sham (no scaffold). Weekly radiographs were taken, and endpoint analysis included micro-CT and histological staining. After 2 weeks of implantation, the BMP-2+ scaffolds were infiltrated with cartilage and woven bone at the non-union site, while GFP+ scaffolds had woven bone formation. Later, timepoints of 8 weeks had woven bone and vessel formation within the BMP-2+ and GFP + scaffolds with cortical bridging of the original fracture site in both groups. Overall, the cell-seeded cryogels promoted osseous healing. However, while the addition of BMP-2 promoted the endochondral ossification, it may provide a slower route to healing. This proof-of-concept study demonstrates the potential for cellularized cryogel scaffolds to enhance the healing of non-unions.

## Introduction

Non-union is defined as the permanent failure of a bone to heal and occurs clinically in approximately 5% of all fractures (Einhorn, 1995; Choi et al., 2004). With increased risk due to factors such as sex, smoking, and diabetes, currently available invasive surgical treatments result in major physical and financial burdens on individuals (Zura et al., 2016; Mills et al., 2017; Dailey et al., 2018; Ekegren et al., 2018). Among the several types of non-unions, atrophic non-unions, characterized by absent/minimal osteochondral callus formation, are poorly understood and difficult to treat (Mills and Simpson, 2012; Thomas and Kehoe, 2021). Furthermore, in atrophic non-unions, bone healing has become stagnant, and surgical intervention with the addition of a biological agent, such as a bone graft, is often necessary to induce regeneration (Moghaddam et al., 2016; Sun et al., 2019). While autografts remain the gold standard for treatment, there are inherent disadvantages including potential for infection, donor site morbidity, and limited availability (Roberts and Rosenbaum, 2012; Sohn and Oh, 2019).

Animal models of atrophic non-unions have been described in mice (Choi et al., 2004; Lu et al., 2007; Garcia et al., 2008; Garcia et al., 2011; Wang et al., 2019), rats (Cullinane et al., 2002; Harrison et al., 2003; Kokubu et al., 2003; Reed et al., 2003; Schmidhammer et al., 2006; Kaspar et al., 2008; Tawonsawatruk et al., 2014; Roberto-Rodrigues et al., 2015), and rabbits (Oni, 1995; Brownlow and Simpson, 2000; Park et al., 2002). Most available models target fracture healing by disrupting the tissues needed for repair through invasive methods such as osteotomy, periosteal stripping, and/or devascularization (Mills and Simpson, 2012; Gómez-Barrena et al., 2015; Panteli et al., 2015). While such methods do yield an atrophic non-union, they do not model some clinical atrophic non-unions, which occur without extensive tissue damage and are attributed to “failure of biology.” We recently developed a novel murine model of atrophic non-union in the 3.6Col1A1-tk (Col1-tk) mouse, where dosing with the nucleoside analog ganciclovir (GCV) is used to deplete replicating osteoblast lineage cells (Hixon et al., 2021). Briefly, in proliferating cells that express the thymidine kinase (tk) “suicide gene,” GCV is converted to a toxic nucleotide, which is then incorporated into the DNA. This results in targeted death of dividing tk + cells, which prevents the proliferative expansion needed to form a callus, leading to callus formation failure after the fracture and thus radiographic and biomechanical non-union. Notably, because non-dividing cells are spared, the fracture site is still populated by viable cells including, presumably, skeletal progenitors. Once GCV is withdrawn, there is potential for these resident cells to proliferate and contribute to fracture healing, although our prior work showed that this does not happen spontaneously (Hixon et al., 2021). This biologically relevant model allows for the testing of novel intervention techniques that might reactivate the quiescent native cells to rescue an atrophic non-union.

Tissue engineering scaffolds provide a promising alternative to currently used bone grafts due to their tailorable properties to modify porosity, mechanical integrity, and degradation rate. Cryogels are hydrogel-based scaffolds that have enhanced porosity and mechanical properties due to the addition of a freezing process during fabrication (Henderson et al., 2013; Hixon et al., 2017a; Bakhshpour et al., 2019). Briefly, following the cross-linking of a polymer solution, the mixture is immediately frozen, resulting in the formation of ice crystals throughout the solution. Upon thawing and melting of these ice crystals, the resulting polymer structure is macroporous, sponge-like, and mechanically durable (Henderson et al., 2013; Okay, 2014). Previous studies have demonstrated that cryogels are suitable for the treatment of complex bone defects including critical size defects in both long bones and craniofacial applications (Dumas et al., 2012; Mishra et al., 2014; Hixon et al., 2017b). However, their application to enhance the repair of atrophic non-unions has not been previously investigated. While tissue engineering approaches have an excellent capability to promote both tissue repair and regeneration of bone injury, further modification can improve their applicability in treating more complex bone defects (e.g., atrophic non-unions, where biological healing has been disrupted) through the ability to deliver specific cell types and/or growth factors. Previous studies have developed woven polymer scaffolds capable of mediating stem cell differentiation and cartilaginous extracellular matrix (ECM) formation using a lentivirus-based method (Brunger et al., 2014; Glass et al., 2014). Subsequent work developed scaffold-mediated lentiviral gene delivery of bone morphogenetic protein-2 (BMP-2), a potent osteogenic factor (Rowland et al., 2018). While woven fiber scaffolds are favorable for tissue-engineered cartilage applications, cryogel scaffolds offer a promising alternative for *in situ* bone regeneration due to their macroporous structure and high pore interconnectivity, which allows fluid flow, cell mobility, and angiogenesis (Hixon et al., 2016; Bakhshpour et al., 2019). Specifically, chitosan–gelatin cryogels are composed of natural materials and have been extensively characterized with pore diameters ranging from 30 to 100 μm conducive to cell infiltration (Kathuria et al., 2009). The combination of cryogel scaffolds with a lentivirus-based method holds the potential to induce bone formation and healing (Rodrigues et al., 2013; Liao et al., 2016; Kemençe and Bölgen, 2017). We focused on the local delivery of BMP-2, long recognized as a potent osteoinductive growth factor capable of improving healing of non-unions, fractures, spinal fusions, and dental implants (Lo et al., 2012; Krishnakumar et al., 2017). Previous studies have shown that following the stabilization of a fracture, exogenous BMP-2 induces cartilage formation and can promote endochondral ossification. Furthermore, the periosteum has been shown to be a target of exogenous BMP-2 (Yu et al., 2010). Notably, previous studies have focused on cellular engineering approaches, including the engineering of mesenchymal stem cells (MSCs) to express BMP-2 in combination with vascular endothelial growth factor (VEGF) or mineral-coated microparticles (Kumar et al., 2010; Orth et al., 2017). Such approaches have demonstrated osseous bridging and increased bone formation. Despite this, the treatment of atrophic non-union can be challenging where the tunable delivery of a biological agent, along with a scaffold matrix, is necessary for appropriate, localized treatment to encourage cell infiltration and initiate healing. Additionally, the exclusively uncontrolled delivery of growth factors such as BMP-2, as well as delivery methods without tunability, can have deleterious results such as excessive heterotopic ossification or increased cancer risk (Shi et al., 2013; Skovrlj et al., 2015; Shi et al., 2017). Thus, we used a doxycycline-inducible BMP-2 lentivirus for the tunable and inducible delivery of BMP-2 in an adipose stem cell-seeded scaffold to the non-union site. The viral vector has been shown to have a high transduction efficiency and result in an inducible production of BMP-2 when exposed to doxycycline (Glass et al., 2014; Rowland et al., 2018). In combination with the biologically relevant Col1-tk mouse model of atrophic non-union, this treatment method allows for the testing of a novel intervention technique to rescue fracture non-union. We hypothesize that the tissue-engineered scaffold-mediated gene delivery of a cell population overexpressing the osteogenic transgene BMP-2 will induce osteogenesis at the site of non-union. Previous work has demonstrated that scaffold-mediated gene delivery can induce sustained transgene expression and ECM formation by MSCs (Kokubu et al., 2003; Hixon et al., 2016; Hixon et al., 2017a; de Lageneste et al., 2018), and we anticipate that using this approach with BMP-2 will provide the osteoinductive environment necessary to drive periosteal healing.

## Materials and Methods

### Scaffold Fabrication

Cryogel scaffolds (*n* = 4/group/timepoint) were fabricated based on a previously established protocol (Kathuria et al., 2009). Briefly, 80 mg of low-viscosity chitosan (MP Biomedicals) was dissolved in 8 ml of 1% acetic acid solution (Fisher Scientific). Following dissolution, 320 mg of gelatin (from cold water fish skin; Sigma) was added to the solution and mixed for 1 h. In a separate scintillation vial, 1% glutaraldehyde solution was prepared using 2 ml of 1% acetic acid solution for cross-linking. All the solutions were cooled at 4°C for 1 h. The solutions were mixed by decanting and then immediately poured into precooled 12-well plates (Sigma), such that a thickness of 3–4 mm was achieved, and frozen at −20°C for 18 h. At this time, the plates were thawed at room temperature (RT) water and trimmed into rectangular sheets (6 mm × 12 mm × 1.5 mm). The scaffolds were sterilized in 1% peracetic acid (PAA; Sigma) for 90 min and then rinsed with sterile 1X phosphate-buffered saline (PBS) three times for 10 min (Yoganarasimha et al., 2014; Costa et al., 2015). Following sterilization, all the scaffolds were placed at −80°C for 1 h and subsequently lyophilized (Sentry 2.0 VirTis BenchTop Pro Freeze Dryer; SP Scientific) for 24 h to ensure complete drying. The resulting cryogel scaffolds were macroporous (pore diameters of 30–100 μm) and sponge-like in structure, allowing them to undergo compression without the crack propagation (Brunger et al., 2014; Gómez-Barrena et al., 2015). Additionally, the cryogels were fabricated from the natural materials that render them compatible with cells and supportive of angiogenesis (Glass et al., 2014).

### Lentivirus Production

The doxycycline-inducible lentiviral vector was generated by cloning *BMP2* into the modified TMPrtTA vector (provided by Danos Lab) as previously described (Rowland et al., 2018). To produce the lentivirus, HEK293T cells were co-transfected with either the dox-*BMP2* vector or a constitutive GFP lentiviral vector with a second-generation packaging plasmid psPAX2 (Addgene, No. 12260) and an envelope plasmid pMD2.G (Addgene, No. 12259) by calcium phosphate precipitation (Salmon and Trono, 2007). The lentivirus was stored at –80°C until further use. The functional titer of each virus was determined *via* qRT-PCR to ascertain the number of lentiviral DNA copies integrated into the genome of the transduced HeLa cells (Salmon and Trono, 2007). On the day of transduction, the lentivirus was thawed and concentrated ∼100-fold using Amicon Ultra 100 kDa MWCO filters (Millipore, UFC9100).

### Scaffold-Mediated Lentiviral Transduction

To immobilize the lentivirus on the scaffolds, cryogels (*n* = 4/group/timepoint) were incubated overnight with 0.002% poly-L-lysine (Sigma), washed with PBS, and then incubated with enough concentrated lentivirus to transduce 500,000 or 1 million cells for 1.5 h at 37°C. The scaffolds were rinsed again with PBS, moved to a new low-attachment 24-well plate (Corning), and seeded with 500,000 cells for *in vitro* experiments and 1 million cells for implanted scaffolds. The expansion medium was added to each sample for 1 h after the cell seeding. All mediums were changed 24 h later. The scaffolds were cultured in the expansion medium for six days to facilitate cell infiltration. A subset of scaffolds were then fixed in a neutral-buffered formalin (NBF) for 24 h, placed in a 30% sucrose solution for an additional 24 h, and embedded in an optimal cutting temperature (OCT) compound. Once frozen, the cryogels were cryosectioned at 20 µm and stained with 4′,6-diamidino-2-phenylindole (DAPI) to visualize the cellular infiltration. All other scaffolds remained in culture.

### *In Vitro* BMP-2 Analysis

After 6 days of culture in the expansion medium, the medium was changed to osteogenic medium containing DMEM-HG (Gibco), 10% lot selected FBS, 1% penicillin–streptomycin (Gibco), 50 μg/ml L-ascorbic acid 2-phosphate (Sigma), 10 nM dexamethasone (Sigma), and 10 mM β-glycerophosphate (Sigma). Doxycycline (dox) groups were supplemented with either 0.1 or 1 μg/ml dox (Sigma). Half medium changes were performed every other day for 28 days, and medium samples were collected on days 0, 2, 6, 10, 16, 22, and 28 of the osteogenic culture. The concentration of BMP-2 in the medium (*n* = 4/group/timepoint) was measured by ELISA (R&D).

### Scaffold Micro-CT Analysis

Following 28 days of osteogenic culture, the scaffolds (n = 4/group/timepoint) were scanned using the Bruker SkyScan 1176 micro-CT at 40 V, 600 µA. Micro-CT datasets were reconstructed with NRecon software using a dynamic range of 0.1, ring artifact correction of 11, and beam hardening correction of 20%. Mineralization was quantified *via* calibration against hydroxyapatite phantoms using CT-Analyzer software. Acellular scaffolds soaked in the medium for 28 days served as a control.

### Mouse Lines

**FIGURE 1:**
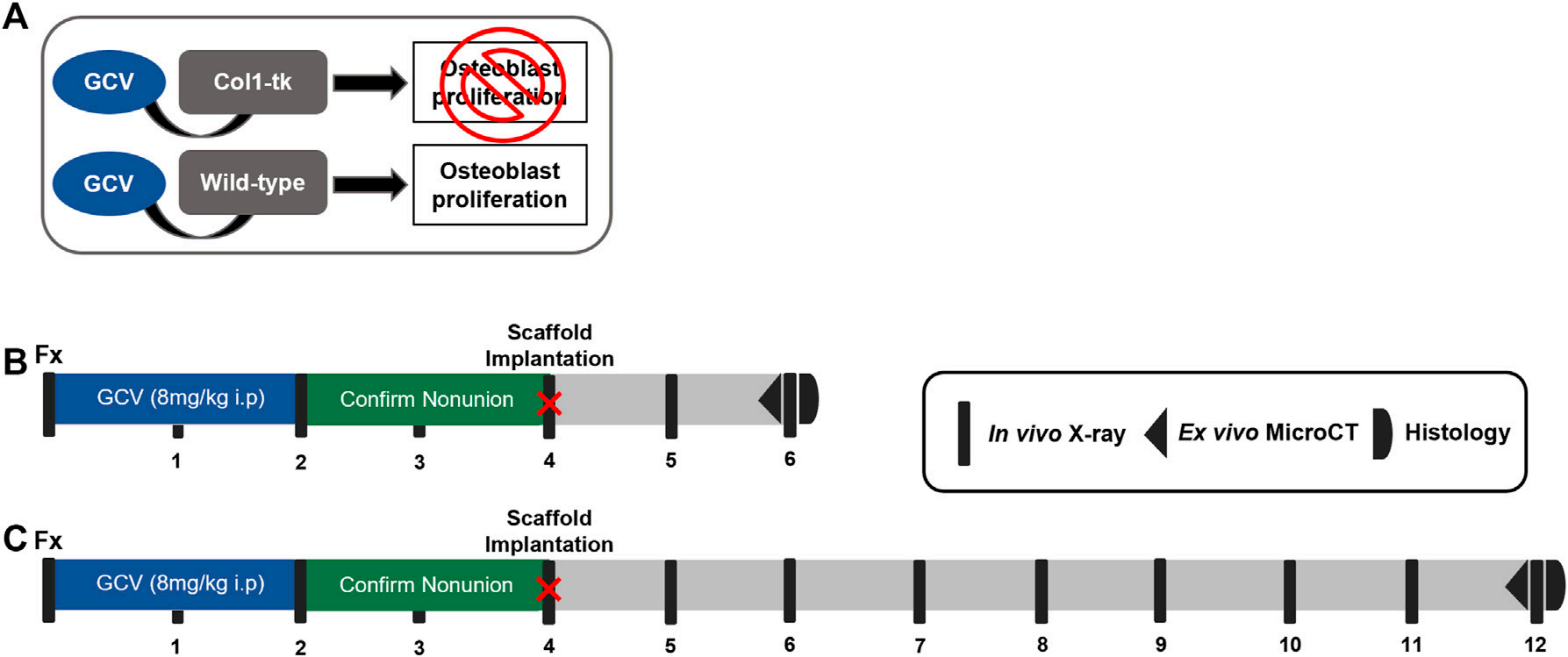
Schematic of the establishment of a non-union and scaffold implantation in Col1-tk mice. **(A)** While receiving the GCV drug, any Col1-tk + cells within the mouse that are proliferating will selectively die. Non-proliferating osteoblasts will remain alive. Once the drug is removed, any proliferating osteoblasts will not die. **(B, C)** Col1-tk mice at 12 weeks of age were subjected to a femur fracture, followed by daily GCV administration for 2 weeks to blunt the healing response. The mice were monitored from week 1–4 by X-ray to confirm minimal callus formation. At 4 weeks post-fracture, an additional surgery was performed, and the scaffolds were implanted. The mice were monitored by X-ray for 2–8 weeks post-scaffold implantation until sacrifice. The outcomes included micro-CT and histology.

A total of 62 male and female mice at 12 weeks of age were used in one of the four experimental groups: sham, scaffold only, GFP cells + scaffold, and BMP-2 cells + scaffold. Experimental procedures were approved by the Institutional Animal Care and Use Committee (IACUC) at Washington University in St. Louis in accordance with the Animal Welfare Act and PHS Policy on Humane Care and Use of Laboratory Animals. Atrophic non-unions were established in transgenic 3.6Col1A1-tk (Col1-tk) mice (rederived from the frozen embryos provided by Drs. Robert Jilka and Charles O’Brien) by targeting the replicating osteoblast progenitors (Jilka et al., 2009). As these mice express the herpes simplex virus thymidine kinase gene (HSV-tk, or “tk”), driven by the 3.6-kb rat Col1A1 promoter which is active in the osteoblast lineage cells; the addition of the nucleoside analog ganciclovir (GCV) targets replicating osteoblast progenitors (Figure 1A). This process occurs through the conversion of GCV to a toxic version of the nucleotide which is incorporated into the DNA, resulting in strands breaking and cell apoptosis (Jilka et al., 2009). To generate Col1-tk mice, male C57BL6/J (The Jackson Laboratory, #000664) mice were bred with female mice, heterozygous for the tk transgene (tk-positive). This resulted in the heterozygous tk-positive (Col1-tk) and tk-negative (wild-type, WT) mice. Only one allelic copy of the tk transgene is necessary to achieve the tk-positive and the male Col1-tk mice are sterile. All genotyping procedures were completed by Transnetyx (Cordova, TN) from toe biopsies taken from the mice for the real-time PCR (probe: puro). Additional breeding was used in the littermates (tk-positive females and tk-negative males). Prior to and during the experimental procedures, all the mice were group-housed (up to five mice/cage) under standard 12-h light/dark cycles with full access to food and water. Note that breeders were given high-fat chow, but following weaning, the experimental mice were given normal chow. All the mice were dosed with ganciclovir (GCV, 8 mg/kg i.p., McKesson) once daily starting at the fracture (12 weeks of age) for 2 weeks. The mice were euthanized by CO_2_ asphyxiation at designated endpoints from 6 to 12 weeks after the fracture.

### Establishment of the Non-Union

We recently demonstrated a model of atrophic non-union in Col1-tk mice dosed with GCV (Hixon et al., 2021). Briefly, a previously established protocol was employed to create a full fracture in the right femur of 12-week-old mice (Gardner et al., 2011). All mice were given buprenorphine SR (1 mg/kg, s.c.) for analgesia 1 h before surgery. The fracture was created by applying a transverse force across the right femoral mid-diaphysis using a custom 3-pt bending setup (DynaMight 8841; Instron). The femur was then stabilized using a 24-gauge metal pin (MicroGroup), and the incision was closed using 3‐0 nylon sutures (Ethicon). We confirmed proper fixation by radiography (Faxitron Ultrafocus 100; Faxitron Bioptics) immediately following the fracture surgery. Note that in the WT mice, this model closely mirrors the classic fracture injury and healing response (Einhorn, 1998; Bonnarens and Einhorn, 1984), displaying both endochondral and intramembranous healing within the fracture gap and around the periphery, respectively (Liu et al., 2017; McKenzie et al., 2019). To establish the atrophic non-union, Col1-tk mice were dosed with GCV at the time of bone injury for 2 weeks, at which time the drug was withdrawn. All the animals were then monitored by weekly radiography (Faxitron) for an additional 2 weeks to confirm a lack of bone formation and healing response, consistent with a non-union (4 weeks following fracture surgery; Figures 1B,C). Dosing, withdrawal, and revision surgery timepoints were based on the previous work in this mouse line and cover the established bridging window in the control mice (Garcia et al., 2013; Liu et al., 2017; Hixon et al., 2021).

### Adipose-Derived Stem Cell Isolation

In the preparation for scaffold implantation, the subcutaneous adipose tissue was dissected from 12-week-old WT mice immediately following euthanasia (CO_2_ asphyxiation). The dissected tissue was minced and placed in a digestion solution (0.1% collagenase and 1% penicillin/streptomycin in Dulbecco’s Modified Eagle’s Medium (DMEM)) at 37°C for 1 h. The samples underwent gentle inversion every 10 min. Following incubation, the tissue was transferred to a 10 cm culture dish, mechanically shredded with forceps, and then placed in an incubator for 30 min (37°C and 5% CO_2_). At this time, the cell suspension was passed through a 70-µm sterile filter and centrifuged at 1300 rpm for 10 min at 4°C. The supernatant was removed and the cell pellet was resuspended in the growth medium (10% fetal bovine serum (FBS) and 1% penicillin/streptomycin in DMEM). All the cells were expanded to passage four which has previously been demonstrated to exhibit greater than 90% homogeneity for the MSC markers (CD13, CD29, CD44, CD73, and CD90) (Mitchell et al., 2006; Baer and Geiger, 2012; Dunham et al., 2020). All the adipose-derived stem cells (ADSCs) were immediately seeded onto the cryogel scaffolds at passage four for the *in vivo* implantation.

### Scaffold Preparation for Implantation

The cryogel scaffolds were fabricated as described previously in the *Scaffold Fabrication*. Three scaffold groups were prepared for implantation: scaffold only (acellular; no lentivirus), scaffold + ADSCs transduced to express GFP, and scaffold + ADSCs transduced to express BMP-2 (*n* = 5–7/group/timepoint). In the preparation for implantation, all the scaffolds were seeded with ADSCs (± lentiviral transduction). Briefly, for the lentiviral transduction, BMP-2 or GFP lentiviruses were immobilized on the scaffold and 1 million cells were seeded and transduced, as described previously in *Scaffold Mediated Lentiviral Transduction*. All samples were cultured for 6 days to allow for cell infiltration and proliferation to occur throughout the cryogels.

### Scaffold Implantation

At 4 weeks following the fracture, the achievement of an atrophic non-union in Col1-tk mice was confirmed *via* the radiograph. All the mice were given buprenorphine SR (1 mg/kg, s. c.) for analgesia 1 h before the surgery. An 8- to 10-mm incision was created along the previously fractured femur and the muscle was gently separated from the non-union site, exposing 7 mm of the femur. The assigned scaffold type (*n* = 5–7/group/timepoint) was placed under the femur and wrapped around the non-union such that the surrounding muscle held the scaffold in place. The muscle was closed with 4.0 vicryl suture, then the incision was closed using the running subcuticular 6-0 PDS sutures (Ethicon). Immediately following the surgery, radiographs were used to confirm that the non-union site remained. Note that the controls included a sham group where the surgery was completed, but no scaffold was implanted. All the animals received dox water (1 μg/ml in 5% sugar water; Sigma) for the duration of the study, changed weekly. The animals were monitored radiographically for 2 or 8 weeks after the surgery, chosen from the previous studies (Douglas et al., 2012; Hao et al., 2016; Orth et al., 2017), to observe the potential rescue of the non-union site. Following euthanasia, both the right and left femurs were dissected for micro-CT and processed for the histological evaluation (Figures 1B,C).

### Radiographic Evaluation

Lateral radiographs were taken weekly following the full fracture until euthanasia (×3 magnification, Faxitron UltraFocus100; *n* = 5–7/group/timepoint). All the radiographs were blindly scored for the degree of healing using a modified RUST scoring system with both the sides of the fracture scored individually and a max score of 6 total: score = 1 designates only the fracture line is present, score = 2 is visible callus and fracture line, and score = 3 is completely bridged with no fracture visible (Cunningham et al., 2017; Fiset et al., 2018; Thomas and Kehoe, 2021). The mice were excluded from the study if there was a loss of fixation during healing or an atrophic non-union was not achieved by week 4 post-fracture (5 mice total).

### Micro‐Computed Tomography

Following euthanasia, the right and left femurs (*n* = 5–7/group/timepoint) were dissected and immediately fixed in 10% NBF for 24 h. After fixation, all the pins were removed from the bone and *ex vivo* scans were completed using micro-CT on the right and left femurs (VivaCT 40, Scanco Medical AG, Switzerland; 10.5 μm voxel size, 55 kV, 145 μA, 300 ms integration time). For the right fractured femurs, a 600 slice (6.3 mm length and diameter) ROI cylinder was centered at the fracture midpoint and thresholds of 180 and 250 per mile was used for 2 and 8 weeks timepoints, respectively, to segment the total bone (callus + cortical). A high-density threshold of 460 per mile was then used to segment out the original cortical bone of all the right (fractured) femurs. The cortical bone volume was subtracted from the total bone volume of each sample to determine the volume of new callus bone formed. In some samples, the fracture lines were no longer visible due to healing, so the point of fracture was found from the radiographic images at 0–2 weeks post-fracture (femoral head to fracture midpoint distance). For the left (intact) femurs, a 100 slice (1.05 mm) ROI was analyzed to compare the systemic changes due to the surgery or BMP-2 production. Following scanning, all the femurs were decalcified and processed for histology.

### Histological Staining and Analysis

Following micro-CT, the samples were decalcified in 14% EDTA (pH 7.0) for 2 weeks (*n* = 5–7/group/timepoint). Standard paraffin processing was completed where all the fractured femurs were sectioned longitudinally at 5 µm thickness. Slides from all the samples were stained with picrosirius red/alcian blue to visualize the different tissues (mineralized callus, cartilage, and fibrous tissue/other) at the fracture site. Immunohistochemistry (IHC) was also used to localize the endomucin expression to evaluate the presence of vasculature throughout the scaffold, as well as the HSV-tk and GFP expression to identify the role of the tk+ and GFP+ cells in healing, respectively. To accomplish this, the paraffin slides were first deparaffinized in xylene and rehydrated in graded ethanol. Next, incubation with 3% H_2_0_2_ for 5 min blocked the endogenous peroxidases. Note that proteinase K was used for the antigen retrieval for endomucin slides prior to blocking (5 min, 23°C). HSV-tk and GFP endogenous epitopes were blocked with 10% goat serum (abcam—ab7481) in PBS at RT for 1 h. The sections were then incubated with rabbit polyclonal anti-HSV tk (1:100; gift from William Summers, Yale) and rabbit-anti-GFP (1:500; Life Technologies A11122) overnight at 4°C, followed by a secondary polymer-HRP anti-rabbit antibody incubation for 1 h (1:200 Dako P0448). The endomucin staining followed the manufacturer’s instructions (Vectastain Elite ABC kit, Rat IgG PK-6104) and used the primary antibodies for endomucin (1:400, eBioV.7C7 #14–5851–85). All the IHC samples were incubated with DAB chromagen (brown; Vector DAB, SK-4105, 30–45 s) and counterstained with modified Mayer’s hematoxylin (Vector Hematoxylin QS, H-3404). Images were obtained on a NanoZoomer slide scanner (20x; Hamamatsu Photonics). Picrosirius Red/alcian blue, HSV-tk, and endomucin images were blinded and qualitatively assessed for the callus and scaffold composition.

### Statistics

Prior to the experiments, sample sizes for the study were calculated using a power analysis with *α* = 0.05 and β= 0.20 (https://www.stat.ubc.ca/∼rollin/stats/ssize/n2.html). The estimates of sample variance and effect size were based on the previous data and biological importance (McKenzie et al., 2019). One-way ANOVA with the Tukey *post hoc* test was used for *in vitro* bone volume and bone mineral density analysis. A chi-square test was used to assess the fracture union based on the RUST radiographic scoring at 2 and 8 weeks post-scaffold implantation. Two-way ANOVA with the Tukey *post hoc* test was used to correct for multiple comparisons for *ex vivo* micro-CT. Significance was considered at *p*-values < 0.05, with trends noted at *p* < 0.10. All the data analysis was performed using Prism (version 9; GraphPad Software, La Jolla, CA, USA).

## Results

### *In Vitro* Analysis Confirms Cellular Infiltration and BMP-2 Production

**FIGURE 2:**
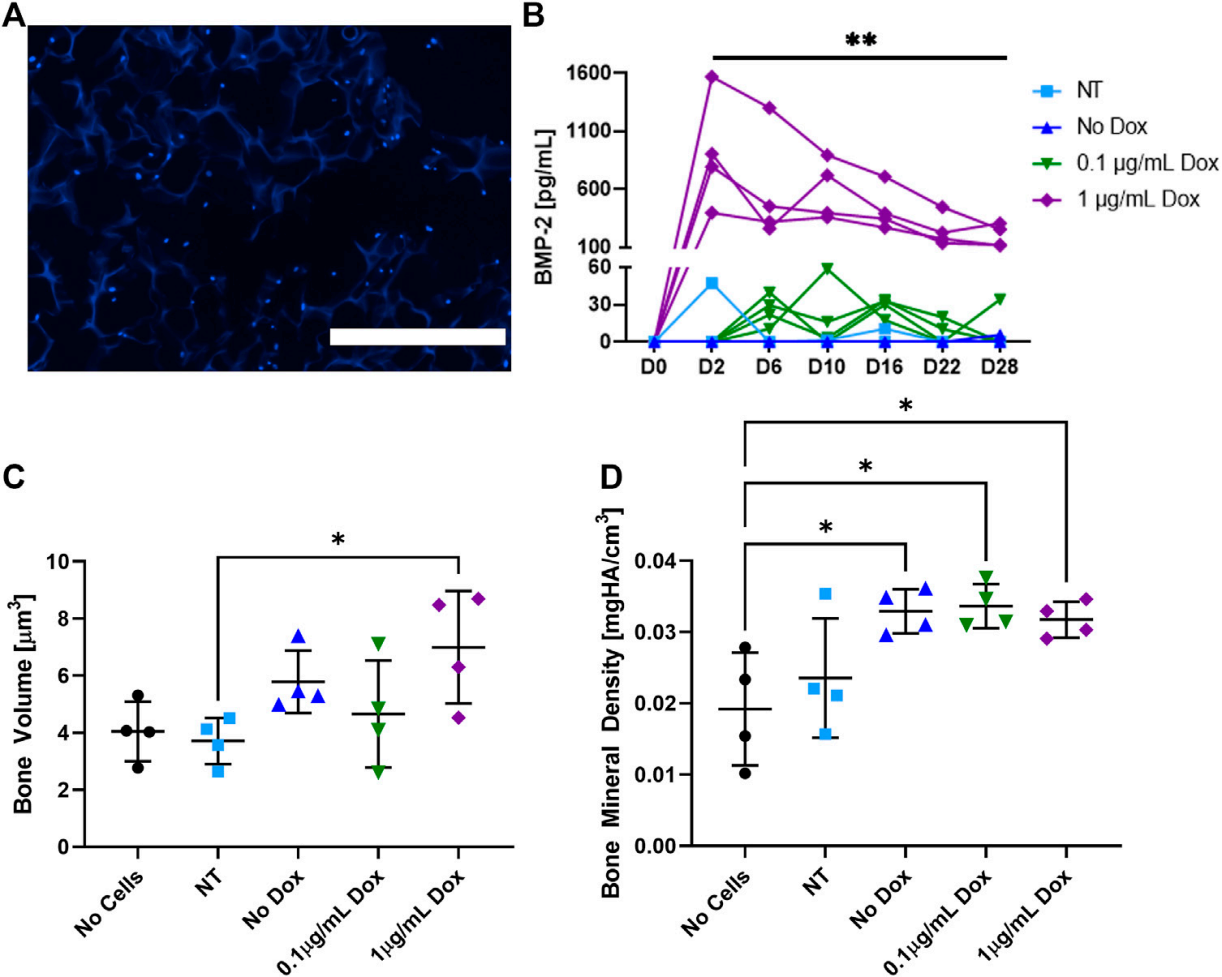
*In vitro* analysis of the cell-seeded cryogel scaffolds. **(A)** Representative DAPI (blue) staining demonstrates the ADSCs infiltrated throughout the cryogel scaffold after 6 days of culture. **(B)** Measured by an ELISA, BMP-2 production was induced in transduced cells treated with 1 μg/ml of dox over 28 days, whereas non-transduced (NT) cells, transduced cells that did not receive dox, and transduced cells that only received 0.1 μg/ml of dox had statistically lower (*p* < 0.01) BMP-2 production. In transduced cells treated with 1 μg/ml of dox, **(C)** mineralized volume and **(D)** bone mineral density were significantly higher than those of non-transduced cells and acellular scaffolds, respectively (*p* < 0.05). The white scale bar denotes 400 µm.

The ADSCs were seeded onto the cryogel scaffolds were cultured for 6 days and then cryosectioned to visualize cell adhesion and infiltration (Figure 2A). As shown by DAPI stain (blue), the ADSCs were able to infiltrate the matrix of all the cryogel scaffolds in the short window of culture, consistent with prior results with other cell types (Hixon et al., 2016; Hixon et al., 2017a). To verify the BMP-2 production, following an initial 6 days of culture, the cells were given osteogenic medium ± the addition of doxycycline (dox; Figure 2B). The non-transduced and transduced cells that did not receive dox (no dox) had negligible BMP-2 production over 28 days of culture. Similarly, a low dose of 0.1 μg/ml of dox was not sufficient to initiate the detectable BMP-2 production. However, when the transduced cells were exposed to 1 μg/ml of dox, a significantly higher production of BMP-2 was noted in all the samples which lasted over 28 days. It should be noted that while there was some variability in the BMP-2 secretion, these are the biological replicates. Thus, the seeded constructs may have been of slightly different sizes, seeded with slightly different numbers of cells, or received slightly different amounts of virus. Following the culture, micro-CT was used to quantify the mineralized volume and density (Figures 2C,D). The transduced cells given 1 μg/ml of dox and thus, producing BMP-2, had significantly more mineralized volume than the non-transduced cells and significantly higher bone mineral density than the acellular scaffolds (*p* < 0.05). Additionally, the transduced cells either left untreated or supplemented with 0.1 μg/ml of dox also had significantly higher bone mineral density than the acellular scaffolds (*p* < 0.05). Note that due to the presence of osteogenic medium, some mineralized volume and bone mineral density levels, when the cells are present, are not unexpected. These results indicate that cryogel scaffolds support mineralization by the ADSCs *in vitro*, and that the mineralized volume is enhanced by the dox-inducible cellular production of BMP-2.

### Radiographic Healing Was Improved by the Presence of Cells

**FIGURE 3:**
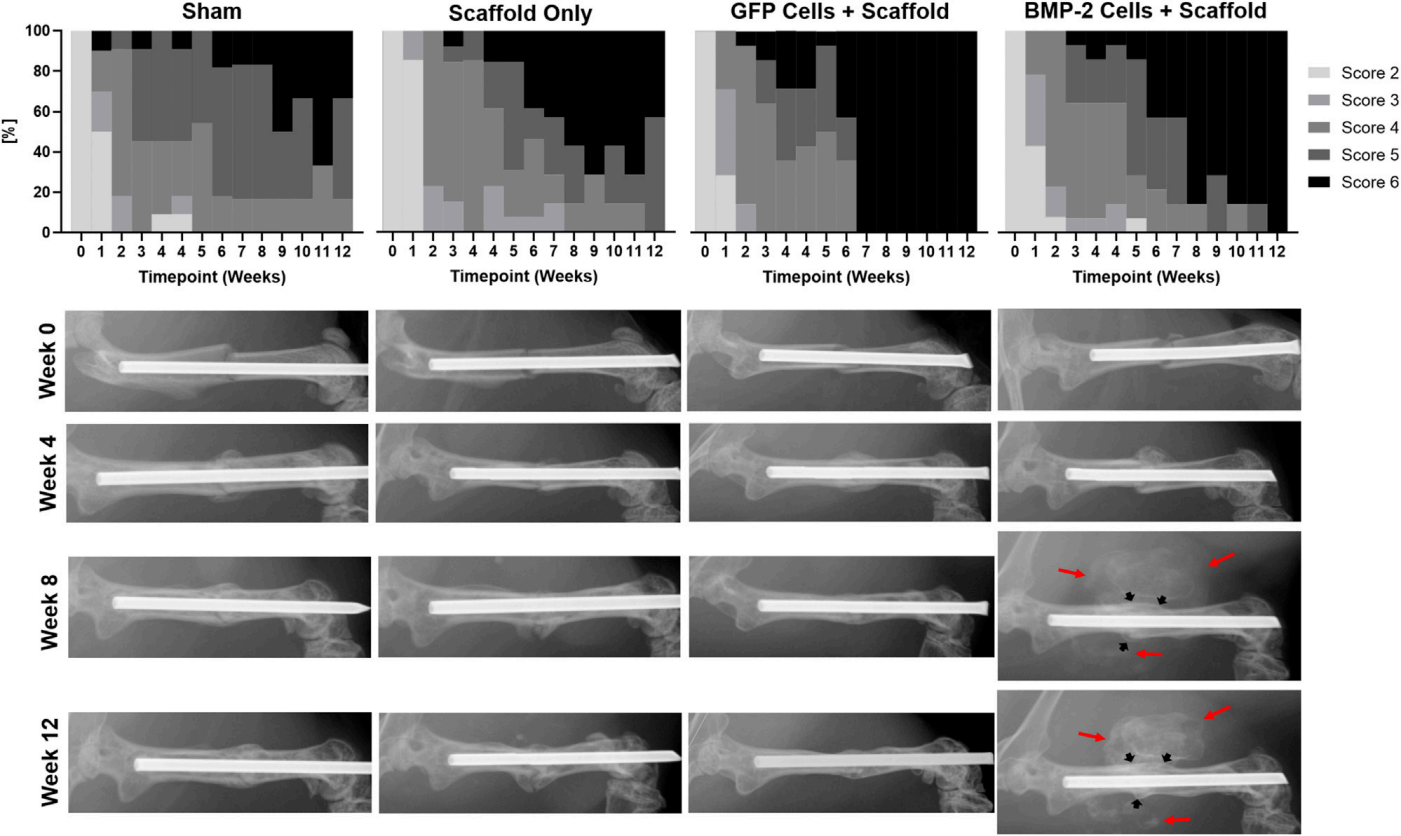
Radiographic scoring of atrophic non-unions treated with sham (no scaffold or cells) control, cryogel (acellular) scaffold only, scaffolds seeded with GFP+ cells, and scaffolds seeded with cells expressing BMP-2. Due to the treatment with GCV and insufficient rescue, less than 50% of the Col1-tk scaffold only or sham groups had complete bridging at 12 weeks. Comparatively, the GFP+ and BMP-2+ cells were the only groups to achieve full bridging by week 12, with GFP+ cells displaying a score of 6 by week 6. Scoring of the radiographs demonstrated no significant difference between any groups at 6 weeks. However, there were significant differences between both the sham and scaffold groups as compared to both the GFP and BMP-2 groups at 12 weeks (χ^2^, *p* < 0.05). Representative radiographs are shown from the time of fracture (Week 0) and at 4, 8, and 12 weeks. The BMP-2+ cellularized scaffolds possessed visually mineralized scaffolds surrounding the non-union site beginning 1–3 weeks following implantation (red arrows) in addition to the endogenous fracture callus (black arrows).

Blinded scoring of the radiographs was completed weekly until sacrifice at 2 or 8 weeks post-scaffold implantation (6 or 12 weeks post-fracture; Figure 3). Less than 50% of the animals treated with either scaffold only or with a sham implantation procedure showed complete bridging of both the cortices at the fracture site at 12 weeks. As both the groups consisted of Col1-tk animals treated with GCV, they were not expected to heal. In comparison, the scaffolds seeded with GFP+ cells and the scaffolds seeded with cells producing BMP-2 were the only groups that reached the complete bridging in all the animals by 12 weeks. Specifically, the fractures in all the animals treated with GFP+ scaffolds had complete bridging (RUST score = 6) after 6 weeks, while over 70% of the BMP-2+ scaffolds were bridged by week 8 and 100% by week 12. Notably, in all the animals treated with the BMP-2+ scaffolds, mineralization was observed radiographically by week 5–7 (1–3 weeks following implantation) throughout the implanted scaffold surrounding the fracture site. In comparison, the GFP+ groups only exhibited mineral within the scaffold in 2 of the 14 samples at varying timepoints. There was no significant difference between the radiograph scores of any groups at 6 weeks. However, the radiograph scores of both the scaffold and sham groups were significantly different than the radiograph scores of both the GFP and BMP-2 groups at 12 weeks. Note that there was no significance between the sham and scaffold groups or the GFP and BMP-2 groups at this timepoint (χ2, *p* < 0.05). In summary, the cellularized scaffolds promoted the radiographic healing, and the addition of BMP-2 stimulated the scaffold mineral formation.

### Non-Unions Treated With BMP-2+ Cellularized Scaffolds Had Higher Mineral Content

**FIGURE 4:**
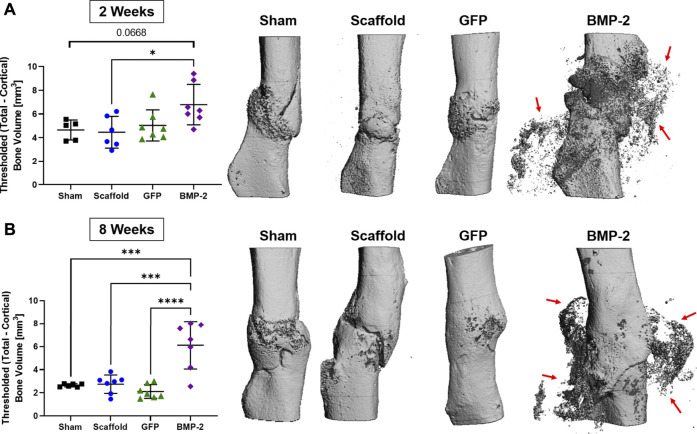
*Ex vivo* micro-CT analysis of non-unions treated with sham control, scaffold only, GFP+ cellularized scaffolds, and BMP-2+ cellularized scaffolds at **(A)** 2 and **(B)** 8 weeks after repair. The callus bone volume is the threshold total bone—cortical bone. At 2 weeks, the BMP-2+ scaffolds had significantly more callus bone volume than (acellular) the scaffold only (*p* < 0.05) and trended higher than the sham control (*p* < 0.0668). At 8 weeks, the BMP-2+ scaffolds had significantly more callus bone volume than all the other groups (*p* < 0.001). Three-dimensional reconstructions demonstrate that fracture lines are still present in the sham and scaffold groups at both 2 and 8 weeks, whereas the GFP and BMP-2 groups exhibited increased bridging and remodeling after 8 weeks. Finally, both timepoints exhibited a high mineral content surrounding the non-union site of the BMP-2+ groups (red arrows).

Following the treatment with scaffolds for 2 or 8 weeks, the animals were sacrificed for micro-CT and histological evaluation. *Ex vivo* micro-CT analysis determined the callus bone volume at both 2 and 8 weeks for all the groups (Figure 4). Note that the 2 and 8 weeks timepoints had a threshold at 180 and 250 per mile, respectively, to isolate the scaffold mineral and thus, cannot be directly compared. At 2 weeks, the BMP-2+ cellularized scaffolds had significantly more bone volume than the acellular scaffold group (*p* < 0.05) and also trended higher compared to the sham control (*p* < 0.0668; Figure 4A). Three-dimensional reconstructions further demonstrate this, with the mineral formation around the non-union site and infiltrating into the surrounding scaffold. At 8 weeks, the BMP-2+ cellularized scaffolds had significantly more bone volume than all the other groups (*p* < 0.001; Figure 4B). Three-dimensional reconstructions at this timepoint also demonstrate mineral formation around the non-union site within the surrounding scaffold. Additionally, while the fracture line remains apparent between 2 and 8 weeks in the majority of the scaffold only and sham groups, the GFP+ and BMP-2+ cellularized scaffolds exhibited a higher ratio of bridging and remodeling of the cortical bone between 2 and 8 weeks. We assessed the skeletal phenotype of the left (intact) femora using micro-CT and found no significance between any groups (*p* < 0.05; Supplementary Table S1), indicating no systemic differences between the treatments. In summary, the cellularized scaffolds promoted bridging at the fracture site, but only BMP-2+ scaffolds produced a significant new bone surrounding the fracture site.

### Histology Demonstrated Vessels Throughout the Surrounding Scaffold and Complete Cortical Bridging With the Addition of Cellularized Scaffolds

**FIGURE 5:**
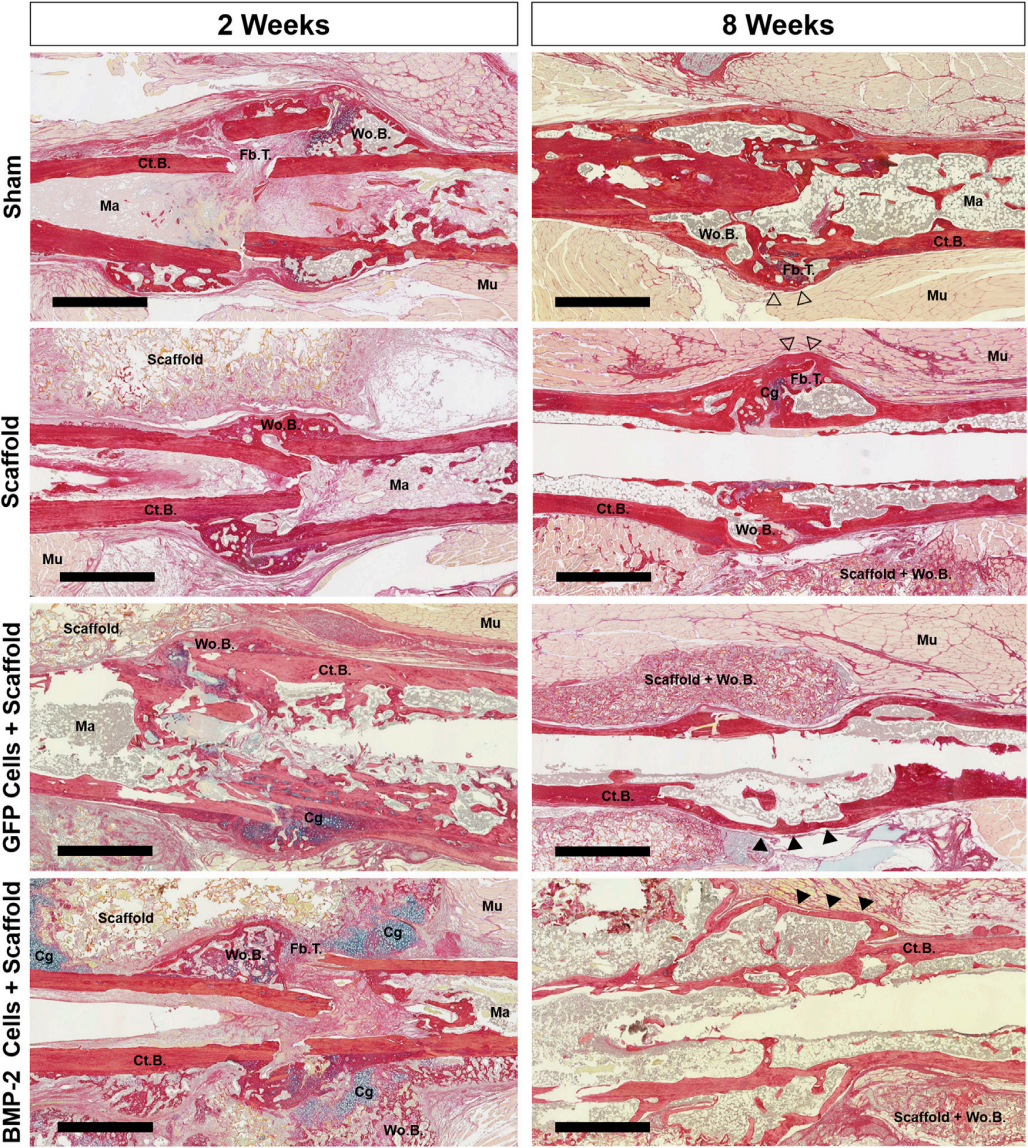
Representative picrosirius red/alcian blue staining of the nonunion site of all groups at 2 and 8 weeks postimplantation. Both the scaffold only and sham groups displayed early woven bone and fibrous tissue, followed by additional bridging and bone formation between 2 and 8 weeks. At 8 weeks, the callus had some pockets of woven bone at a still visible fracture site (open arrowheads). While the GFP+ group had some cartilage, it was minimal and resulted in bridging in all animals by 8 weeks. At 2 weeks, 6 of the 7 BMP-2+ groups displayed the cartilage both surrounding the nonunion site and within the surrounding scaffold. After 8 weeks, bridging was visually achieved in all BMP-2 samples with mineral infiltration into the surrounding scaffolds. Cortical bridging was denoted by filled arrowheads. Scale bars denote 1 mm. Cg, cartilage; Ct.B, cortical bone; Fb.T, fibrous tissue/other; Ma, marrow; Mu, muscle; Wo.B, woven bone.

Following the micro-CT analysis of 2-8 weeks samples, histological evaluation was completed using picrosirius red/alcian blue stain (Figure 5). At 2 weeks post-sham surgery (6 weeks post-fracture), the sham control group displayed minimal woven bone formation and high levels of fibrous tissue. After 8 weeks, incomplete healing was still noted in 5 of the 6 samples, despite woven bone formation and some cortical bridging at all of the fracture sites. Similarly, the scaffold only treatment displayed some calcified areas with almost no cartilage present and minimal infiltration into the surrounding scaffold, showing incomplete endochondral bone formation. After 8 weeks, full bridging (denoted by continuous cortical bone) had visually occurred in 3 of the 7 samples, with the remaining samples possessing fibrous tissue and minimal cartilage. In the animals treated with the GFP+ cellularized scaffolds, new bone was formed at 2 weeks with the presence of minimal cartilage and fibrous tissue. By 8 weeks postimplantation, full bridging was achieved in 7 of the 7 samples, with the new bone infiltrated into the surrounding scaffold and no fibrous tissue or cartilage. When the cryogel scaffolds seeded with the BMP-2-expressing cells were implanted, cartilage was visualized surrounding the non-union site and within the surrounding scaffold in 6 of the 7 samples at 2 weeks. After 8 weeks, bridging was visually achieved in all the samples with the mineral infiltrating any surrounding scaffold still present; no fibrous or cartilage callus tissue was observed. In summary, the majority of samples in the sham and scaffold only groups displayed remnants of the original fragmented cortical bone with visible fracture surfaces and lacked complete bridging, whereas the GFP+ and BMP-2+ groups displayed continuous cortical surfaces such that the fracture line was no longer visible in most samples.

**FIGURE 6:**
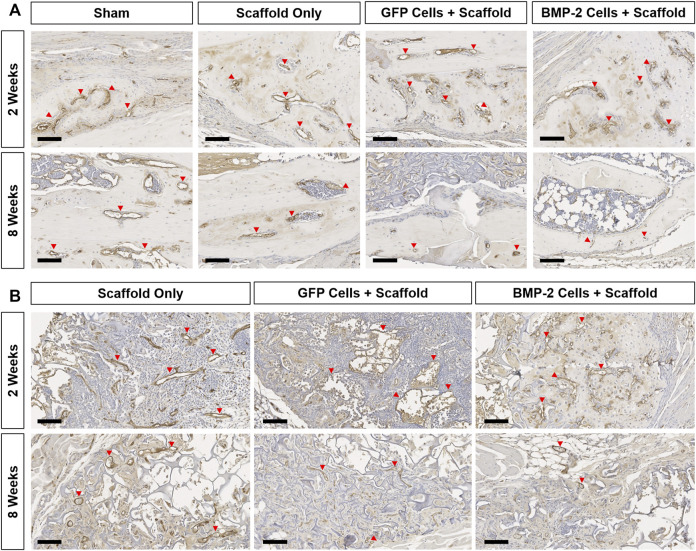
Representative endomucin (brown) staining of the vasculature within the non-union and scaffold sites of all the groups at 2 and 8 weeks postimplantation. **(A)** 10x images of the woven bone at 2 weeks show extensive vessel staining in all the groups (red arrows). Comparatively, at 8 weeks, vessel staining was greatly reduced in all groups. **(B)** Endomucin staining within the scaffold showed vasculature associated with new bone formation at both the timepoints in all groups treated with a scaffold ± cells. Scale bars denote 100 μm.

The sections were also stained for endomucin to identify the presence of vasculature, HSV-tk to localize the tk + cells, and GFP to discern the role of GFP+ cells in both the endogenous callus at the non-union site, as well as within the scaffold (Figure 6, Supplementary Figures S1, S2). Overall, all the groups possessed a large number of vessels directly at the fracture site and closely associated with the woven bone formation at 2 weeks (Figure 6A). After 8 weeks, the vasculature at this site was greatly reduced with little to no endomucin staining in all the groups. When the surrounding scaffolds were examined (Figure 6B), the vessels were visible both within the scaffold matrix and accompanying the new bone formation at 2 and 8 weeks in all the groups treated with a scaffold ± cells. The new bone was typically formed on the edges of the scaffold, both proximal and distal to the fracture site. With respect to the HSV-tk staining, the positively stained osteoblasts and osteocytes were detected within the bone tissue in some samples from every group at both 2 and 8 weeks (Supplementary Figure S1). After 8 weeks, all the BMP-2+ scaffolds also possessed HSV-tk + cells (red arrows) localized to the new bone formed within the scaffold, indicating the invasion of host cells. Finally, GFP+ staining was found within the scaffold around the fracture site at 2 weeks in all the samples treated with scaffolds seeded with GFP+ cells (Supplementary Figure S2). However, after 8 weeks, there was no GFP+ staining in 5 of the 7 samples, with very little staining in the remaining 2 samples, demonstrating that the GFP+ cells were no longer present at 8 weeks in the majority of samples.

## Discussion

We hypothesized that a bioactive cryogel-mediated, doxycycline-inducible lentiviral gene delivery system of BMP-2 would induce osteogenesis at the atrophic non-union defect site. This approach was based on the previous studies which demonstrated that scaffold-mediated gene delivery can induce the sustained transgene expression and protein synthesis by MSCs (Brunger et al., 2014; Glass et al., 2014; Moutos et al., 2016; Huynh et al., 2018; Rowland et al., 2018). Overall, the *in vitro* data demonstrated the feasibility of the cryogel-mediated lentiviral delivery of BMP-2 with adhesion and infiltration of cells, as well as the sustained BMP-2 production over 28 days. *In vivo*, following implantation at 4 weeks, 50% or less of the animals treated with either scaffold only or the sham control had a complete radiographic bridging at 12 weeks. This blunted healing response was consistent with our previous study that established the Col1-tk atrophic non-union model (Hixon et al., 2021). Comparatively, the radiograph analysis showed that the scaffolds seeded with GFP + cells and the scaffolds seeded with cells producing BMP-2 were the only groups that reached complete bridging in all the animals by the end of 12 weeks. Mineralization was also noted radiographically throughout the implanted scaffold surrounding the fracture site in all the animals treated with the BMP-2+ cells. Histologically, it was observed that the GFP+ and BMP-2+ scaffolds both had woven bone formation as early as 2 weeks after the implantation, with BMP-2+ scaffolds displaying both cartilage and woven bone directly at the non-union site, as well as infiltrated into the surrounding scaffold. At 8 weeks after the GFP+ and BMP-2+ scaffold implantation, the woven bone was noted within the scaffold and the non-union site had undergone complete bridging as noted by the continuous cortical bone. Furthermore, the overall addition of a scaffold ± cells resulted in blood vessel formation, as well as HSV-tk + cells in the BMP-2 samples, localized to new bone within the scaffold matrix at 8 weeks. Importantly, in the GFP+ cell group, while the GFP+ cells were noted histologically at 2 weeks, the majority of samples had no GFP+ cells within the scaffold or around the fracture site at 8 weeks. This observation indicates that the native cells were responsible for bone formation seen at 8 weeks, and we infer that the native cells that were not targeted during the 2 weeks of GCV dosing were activated by the implantation of the cellularized scaffolds. Overall, the cell-seeded cryogels resulted in osseous healing and while the addition of BMP-2 promoted endochondral ossification, this appeared to delay healing contrary to our initial hypothesis. It is possible that the promotion of endochondral ossification may produce a better functional result in the long term as it mimics normal fracture healing. However, this should be further investigated with future biomechanical testing. Our findings support further investigation and development of a cell-seeded tissue engineering cryogel (±BMP-2) to target specific timepoints in the clinical non-union healing process.

Endochondral ossification plays a critical role in fracture repairing and remodeling. This process occurs between the two fractured ends where the cartilage contributes to a soft callus to initially stabilize the fracture site. In combination with intramembranous ossification and the formation of a hard callus, full bridging occurs with the continuous cortical bone (Marsell and Einhorn, 2011). BMP-2 has been previously shown to induce chondrogenic differentiation, osteogenic differentiation, and endochondral ossification within the same system (Zhou et al., 2016). Thus, the expression of BMP-2 not only encouraged bone formation within the implanted scaffolds surrounding the fracture site, but also appeared to promote endochondral ossification and bridging at the fracture site. Notably, cellularized scaffolds without the BMP-2 transfection also appeared to improve healing at the fracture site, and thus we are not able to fully attribute the improved healing to the effects of local BMP-2 production based on this initial study. Future work should examine shorter timepoints to identify the windows of healing as guided by the addition of the scaffold-mediated lentiviral delivery of BMP-2. This would allow for targeted healing within an atrophic non-union. Additionally, the scaffold-mediated lentiviral transduction technique could be applied to an acellular scaffold to deliver BMP-2 to the non-union site, encouraging a similar endochondral ossification response without using implanted cells.

The use of the Col1-tk mouse as a model of atrophic non-union, as well as our scaffold fabrication and implantation techniques, has several limitations. Our previous study has demonstrated that 2 weeks of GCV dosing in Col1-tk mice blunted callus formation and led to a functional non-union at 12 weeks (Hixon et al., 2021). We also observed that *via* radiography, *in vivo* micro-CT, and histology that the fracture healing response did not change after the first 4 weeks in Col1-tk mice, that is, once the non-union was established, it did not recover on its own. Thus, we selected the 4-weeks timepoint for the intervention in the current study. Unexpectedly, we noted that in some sham and scaffold only samples, there was radiographic and histological evidence of cortical bridging. It is possible that the surgical intervention increased blood flow at the injury site or otherwise created an environment that reactivated periosteal cells that were dormant. In addition, the previous work has demonstrated that muscle-derived stem cells can differentiate into the cartilage and bone and directly participate in fracture healing (Liu et al., 2010; Shah et al., 2013). A short period of GCV dosing following the scaffold implantation surgery may block the healing due solely to surgical intervention, providing a more challenging environment to test the interventions. Regarding the cryogel scaffolds, these were designed for the ease of handling and cell seeding *in vitro,* as well as for the feasibility of surgical implantation. Yet, the thickness of the scaffolds was large relative to the femur dimensions, and they showed some tendency to “unwrap” after the implantation. This resulted in a diffuse region of mineralization in the BMP-2+ scaffold group. Additionally, the thick scaffold layer may have hindered the cellular and vascular infiltration of the scaffold, as well as integration of the scaffold bone with the native callus at the bone surface. Despite this, 5 of the 7 BMP-2 group had mineralized scaffold directly integrated with the fully bridged cortical bone. In the remaining 2 samples, the mineralized scaffold rested against the bridged fracture site. However, the cortical bone was continuous with no apparent fracture sites, demonstrating the overall contribution to healing by the BMP-2+ scaffolds as compared to scaffold only. Future studies will further optimize the scaffold size and overall placement to minimize disruption and physical scaffold movement following implantation. Finally, this study served as a proof-of-concept, and overall, BMP-2 delivery was not controlled or localized appropriately. Future work should reconfigure the release to be more localized to the fracture site and target the BMP-2-induced endochondral ossification, which could be advantageous to bone healing. In addition to this, it was demonstrated that the dox-inducible BMP-2 expression can promote endochondral ossification in the cryogel itself which could be optimized spatially and temporally.

The current clinical practice of non-union often involves surgical intervention and the use of autologous bone graft. While the autologous graft is effective in promoting healing, it requires a second-site surgery and has other limitations/contraindications including potential infection, donor site morbidity, and limited availability (Roberts and Rosenbaum, 2012; Sohn and Oh, 2019). Therefore, we selected a tissue engineering approach that does not use the autologous bone graft, but does require a surgical intervention, as a first step toward an alternative to current practices. Of interest was the finding that the cell-seeded scaffolds may enhance the healing in atrophic non-unions. Notably, the sham group histologically closely mirrored the delayed bone bridging and blunted callus formation as previously shown in our Col1-tk mouse model of atrophic non-union where no intervention had been employed (Hixon et al., 2021). In contrast, both the GFP+ and BMP-2+ cellularized cryogel scaffolds resulted in the radiographic bridging at the site of the non-union. The GFP+ scaffolds appeared to result in faster healing than the BMP-2+ scaffolds. This could be attributed to the initial high cartilage response in the BMP-2+ scaffolds, which may have delayed the overall healing of the non-union. One of the major downfalls in treating complex bone defects is a lack of structure and stability to guide healing. To combat this, tissue engineering scaffolds provide a unique template that can be tailored to target an appropriate structure. Combining the tunable delivery of a biological agent with a scaffold may further enhance healing in complex atrophic non-unions. In this study, the addition of cells in combination with the cryogel scaffolds resulted in cortical bridging, suggesting that cryogels seeded with the ADSCs (with or without the addition of BMP-2) is a promising experimental approach to promote the rescue of the non-union. Future work should include mechanical testing to directly compare BMP-2+ and GFP+ cellularized scaffolds to define the functionality of this rescue technique. Additionally, as this is a tunable/inducible system, the BMP-2 delivery window should be further explored to identify if it is advantageous to include BMP-2 or if solely providing the cells is sufficient to induce regeneration.

In conclusion, we demonstrated that a cryogel scaffold seeded with cells enhances fracture healing at a non-union site as radiographic and histological bridging was achieved in all animals treated with cell-seeded scaffolds (±BMP-2). Furthermore, the addition of BMP-2 resulted in cartilage formation at early timepoints, stimulating the process of endochondral ossification, but slightly delayed overall healing. Overall, the cryogel scaffolds provided a supportive, porous matrix for bone ingrowth and new tissue formation. This research is significant as it utilizes a clinically relevant model of atrophic non-union to evaluate a novel rescue technique to induce bone formation and promote healing at the non-union site. These promising initial findings support the continued refinement and the study of cellularized cryogels for treating the non-healing fractures.

## Data Availability

The raw data supporting the conclusion of this article will be made available by the authors, without undue reservation.
