# Supplementary material for: Cryogel Scaffold-Mediated Delivery of Adipose-Derived Stem Cells Promotes Healing in Murine Model of Atrophic Non-Union
Source: Front Bioeng Biotechnol. 2022 May 5;10:851904. doi: 10.3389/fbioe.2022.851904 (PMC9117654; doi:10.3389/fbioe.2022.851904)
Supplement: Supplementary file 2 [file DataSheet1.docx]

**
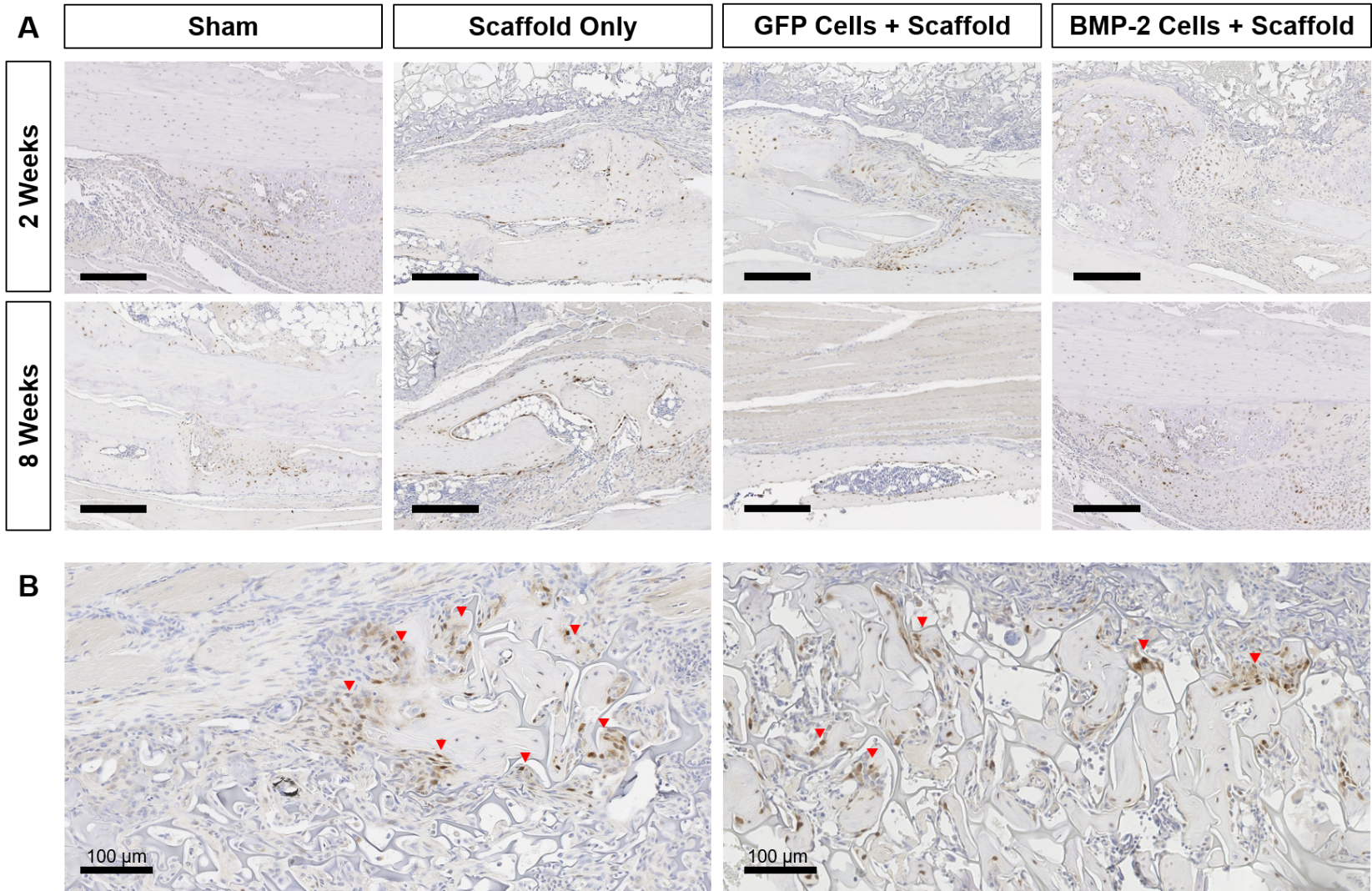
**

**Supplementary Figure 1.** Histological characterization of HSV-tk+ cells within the (A) fracture callus and (B) surrounding scaffold. At both 2 and 8 weeks, HSV-tk+ cells (stained brown) were predominately detected within bone tissue (osteoblasts and osteocytes) in some samples from each group and timepoint. (B) After 8 weeks, HSV-tk+ cells (red arrows) were also found to be localized to the new bone formed within the scaffold of all samples treated with BMP-2+ scaffolds. Black scale bars denote 250 µm unless otherwise noted.


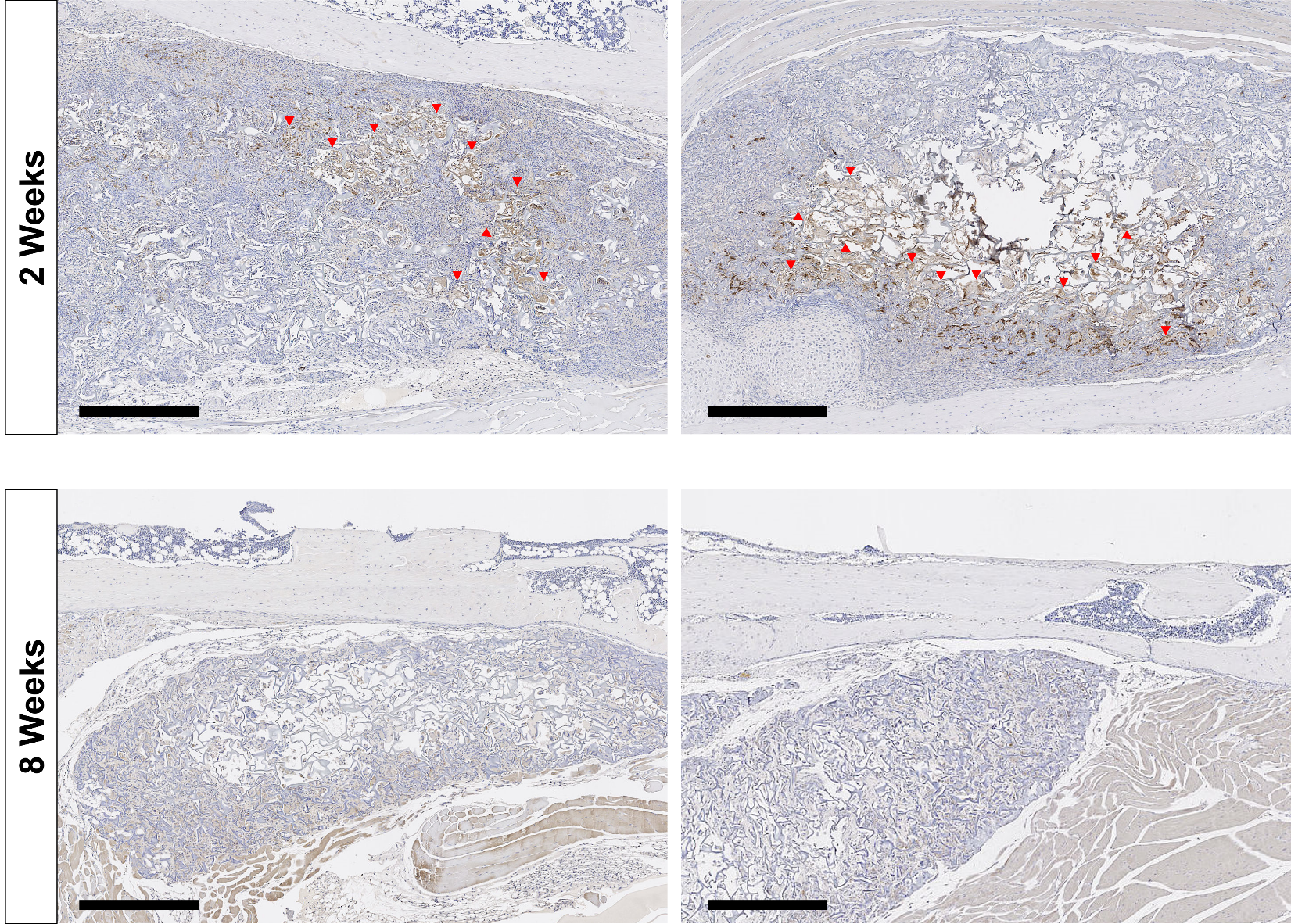


**Supplementary Figure 2.** Histological characterization of GFP+ cells within the surrounding scaffold of two different samples at 2 and 8 weeks. In all 2 week samples, GFP+ cells (stained brown) were detected throughout the scaffold along the fracture callus. However, after 8 weeks, 5 of the 7 samples had no staining and the remaining 2 samples had very minimal staining. Black scale bars denote 500 µm.
